# Neuropsychological performance differences between two groups of probable-AD patients from different areas of Brazil

**DOI:** 10.1590/S1980-57642012DN06020006

**Published:** 2012

**Authors:** Analucy Aury Vieira de Oliveira, Corina Satler, Carlos Tomaz

**Affiliations:** 1 PhD candidate, Laboratory of Neuroscience and Behavior, Department of Physiological Sciences, Institute of Biology and Graduate Program in Health Sciences, Faculty of Health Sciences, University of Brasília, Campus Darcy Ribeiro, Brasília DF, Brazil.; 2 PhD, Laboratory of Neuroscience and Behavior, Department of Physiological Sciences, Institute of Biology, University of Brasília, Campus Darcy Ribeiro, Brasília DF, Brazil.; 3 Full Professor, Laboratory of Neuroscience and Behavior, Department of Physiological Sciences, Institute of Biology, University of Brasília, Campus Darcy Ribeiro, Brasília DF, Brazil.

**Keywords:** dementia, cognitive impairment, Alzheimer's disease, neuropsychological assessment, diagnosis

## Abstract

During normal aging there are some cognitive and behavioral changes similar to those observed in a transitional state or mild cognitive impairment (MCI) and early onset dementia, making it challenging for health care professionals to reach an accurate and reliable diagnosis.

**Objective:**

The current study examined the performance of two different groups of patients diagnosed with probable Alzheimer's disease (AD) on a neuropsychological test battery.

**Methods:**

Twenty-two AD patients from Brasília-DF (AD1) and thirty-four AD patients from Palmas-TO, northern Brazil (AD2), were selected and a short neuropsychological battery administered. To verify the reliability of these previous diagnoses of AD, both groups of patients were compared with a group of healthy controls.

**Results:**

AD patients showed cognitive deficit but scores were lower for the AD2 group compared with the AD1 group considering the cut-off point. Notably, patients from the AD1 group were older (p=0.004) and had less formal education (p<0.001) than those from the AD2 group. Comparing different cognitive domains between AD groups, post hoc analysis showed that the AD1 group was characterized by deficits in episodic memory retrieval (p<0.001), semantic memory (p<0.001) and verbal fluency (p<0.001). In contrast, the AD2 group showed lower scores in attention (p=0.007), executive functioning (p<0.001) and working memory (p<0.001).

**Conclusion:**

This pattern suggests that the Palma group of patients had a neuropsychological profile that was inconsistent with AD. Although the results of this study have important clinical implications, the effects of age, education, and gender on cognitive performance should be explored further.

## Abstract (pt)

Durante o envelhecimento normal existem algumas mudanças cognitivas e comportamentais similares àquelas observadas no estágio transicional ou declínio cognitivo leve e demência precoce, desafiando os profissionais da saúde a fazer um diagnóstico preciso e confiável.

**Objetivo:**

O presente estudo investigou o desempenho cognitivo de dois diferentes grupos de pacientes com provável diagnóstico de doença de Alzheimer (DA).

**Métodos:**

Vinte e dois pacientes de Brasília-DF (DA1) e trinta e quatro pacientes de Palmas-TO, norte do Brasil (DA2), foram submetidos a uma bateria neuropsicológica reduzida para verificar a confiabilidade do diagnóstico prévio de DA, comparando-se ambos os grupos a um grupo de idosos sadios.

**Resultados:**

Pacientes com DA mostraram déficit cognitivo; no entanto, os escores foram mais baixos para o grupo DA2, considerando-se o ponto de corte. É importante destacar que os pacientes do grupo DA1 foram mais velhos (p=0,004) e com menor nível de educação formal (p<0,001) que o grupo DA2. Quando comparados os diferentes domínios cognitivos entre os grupos com DA, a análise post hoc indicou que o grupo DA1 caracterizou-se por déficits em recuperação de informação episódica (p<0,001), memória semântica (p<0,001) e fluência verbal (p<0,001). Em contraste, o grupo DA2 mostrou escores menores em atenção (p=0,007), funcionamento executivo (p<0,001) e memória operacional (p<0,001).

**Conclusão:**

Estes resultados sugerem que o grupo de pacientes de Palmas apresentou um perfil neuropsicológico não compatível com DA. Embora os resultados deste estudo tenham importantes implicações clínicas, o efeito da idade, educação e gênero no desempenho cognitivo devem ser mais explorados.

## INTRODUCTION

The Brazilian population is aging and the number of elderly people in Brazil is estimated at over twenty million.^1^ One of the major consequences of this growth is an increase in the prevalence of neuropsychiatric pathologies and neurodegenerative diseases.^2^

Dementia can be defined as a clinical condition characterized by cognitive decline leading to significant impairment in patients' activities of daily living, social and occupational performance.^3^

The Brazilian Academy of Neurology (ABN)^4^ recommends that the clinical diagnosis of dementia be based on the criteria of the 4^th^ edition of the Diagnostic and Statistical Manual of Mental Disorders by the American Psychiatric Association (DSM-IV).^5^ In order to be diagnosed with dementia, the individual must present with prior decline in functioning as a result of memory im­pairment, and show impairment of at least one cognitive function: language, agnosia, praxis, executive function or spatial function. Also, these deficits must not occur exclusively during acute confusional syndrome or *delirium* pictures.

Among the different types of dementia, Alzheimer's disease (AD) is the most frequent followed by vascular dementia.^2,6^

Alzheimer's disease is an age-related degenerative brain disorder characterized by neuronal atrophy, synapse loss, and the abnormal accumulation of amyloidogenic plaques and neurofibrillary tangles in medial temporal lobe limbic structures and the association cortices of the frontal, temporal, and parietal lobes.^7^ In order to diagnose AD, the ABN recommends the adoption of the criteria of the *National Institute for Communicative Disorders and Stroke-Alzheimer's Disease and Related Disorders Association* (NINCDS-ADRDA) by McKhan et al. (1984).^8^

Clinical evidence suggests that the first changes occur in medial temporal lobe structures critical for episodic memory,^9^ and consequently episodic memory impairment is usually the earliest and most salient aspect of AD.^7^ Additionally, as the neuropathology of AD spreads further to the association cortices of the temporal, frontal, and parietal lobes,^9^ a number of higher-order cognitive abilities are affected and patients develop a semantic memory deficit in later stages of the disease.^7^

Moreover, several studies have confirmed that AD patients lose their critical judgment as the disease progresses.^10,11^ Memory deficits and impaired reasoning and judgment cause a significant impairment in activities of daily living and affect patients' autonomy and decision-making abilities. The loss of decision-making ability has direct implications regarding patients' medical and legal capacity to make decisions concerning treatment, institutionalization, financial management, and the decision to participate in research studies.^12-14^

The insidious onset of AD shows a significant prodromal phase of varying length, with studies indicating periods of up to 20 years to reach a definitive AD phase. Misdiagnosis is not uncommon when evaluating cognitive decline, as several symptoms of AD can be mistaken for mild cognitive impairment symptoms.^12,15,16^ Thus, the course of AD can be separated into predictable clinical stages ranging from prodromal mild cognitive impairment to moderate and profound dementia.^17^

Therefore, better accuracy in reaching a differential diagnosis is achieved through a combination of a clinical examination including in-depth anamnesis, neurological examination and neuropsychological assessment, with complementary investigation comprising laboratory and neuroimaging exams.^18^

More specifically, neuropsychological batteries are based on a combination of instruments that assess cognitive and behavioral functions. This assessment is important to support the differential diagnosis and prognosis, enabling sound orientation for treatment and planning of rehabilitation.^19^

In Brazil, neuropsychology researchers have studied the performance of the elderly population using different cognitive tests. Cognitive assessment typically starts with application of the Mini-Mental State Examination (MMSE).^20^ This instrument is widely used to screen for cognitive impairment in clinical practice and dementia studies.^21,22^

In cases of poor performance on the MMSE, a more comprehensive assessment is conducted by applying tests that assess multiple cognitive domains. The Mattis Dementia Rating Scale (DRS)^23^ is commonly employed by neuropsychologists in clinical settings.^24^ Currently, there are numerous neuropsychological batteries available that are validated and adapted for use in the Brazilian population (e.g., the Consortium to Establish a Registry for Alzheimer's disease - CERAD,^25^ Cambridge Cognitive Test - CAMCOG,^26^ and the cognitive subscale of the Alzheimer's Disease Assessment Scale - ADAS-Cog^27^).

While definitive diagnosis is only derived from autopsy findings, clinical diagnosis has traditionally centered on cognitive symptoms and exclusion criteria. Hence, AD is regarded as a diagnosis of inclusion, characterized by specific patterns of neuropsychological dysfunction and slow, insidious onset and progression, in which neuropsychological assessment plays an invaluable role as a complement towards reaching a decision on diagnosis. However, such assessments are not always performed.

The aim of this study was to describe the global cognitive profile of two groups of patients diagnosed with AD from two different regions of Brazil (Palmas, Tocantins state and Brasília, Federal District) and to compare the raw scores obtained by study participants with those of a group of elderly without dementia (control group).

## METHODS

**Participants.** This study included 56 patients diagnosed with AD, 22 of whom resided in Brasília [AD1: 15 women] and 34 in Palmas [AD2: 31 women], in addition to 40 healthy elderly adults [elderly controls (EC): 24 women]. Mean age was 78.27±6.70 years for AD1, 72.56±4.09 years for AD2, and 71.10±6.72 years for EC; mean schooling was 6.73±4.00 years for AD1, 11.47±3.33 years for AD2, and13.25±5.57 years for EC.

The Palmas Group was examined by gerontologists, neurologists or psychiatrics and referred to a neuropsychologist for performance testing and evaluation in order to reach a more accurate diagnosis. A clinical diagnosis of AD was determined for each patient at a research team meeting at the University of Brasília-UnB.

The Brasília Group was recruited from the Geriatric Medical Center, University Hospital of Brasília, Brasília, Brazil. All patients underwent examinations by a social worker, neuropsychologist, and geriatrician and a clinical diagnosis of AD was determined for this patient group.

Selections were made in accordance with the clinical diagnostic criteria of AD (National Institute of Neurological and Communicative Disorders and Stroke-Alzheimer's Disease and Related Associated Disorders, NINCDS-ADRDA).^8^

Additionally, the elderly controls group comprised individuals living in the community and nonconsanguineous relatives.

The severity of AD ranged from mild to moderate (scores 1 or 2) according to the Clinical Dementia Rating Scale (CDR).^28^ All patients exhibited a 1- to 4-year history of progressive cognitive impairment predominantly affecting memory, which was confirmed by their caregiver using the IQCODE (Informant Questionnaire on Cognitive Decline in the Elderly),^29^ but showed normal awareness and lived with their families.

The Neuropsychiatric Inventory (NPI)^30^ and Cornell Scale for Depression in Dementia (CSDD)^31^ were applied to all subjects. Whenever evidence of behavioral disturbance or significant depression symptoms was noted on interview, the subject was excluded.

Written informed consent in accordance with the ethical guidelines for research with human subjects (196/96 CNS/MS, Brazil, resolution) was obtained from all participants or their caregivers (where appropriate). The study protocol was approved by the Ethics Committee for Research in Human Subjects of the Faculty of Health Sciences, University of Brasília.

**Neuropsychological assessment.** The neuropsychological evaluation was performed by (C.S) in both AD1 and EC groups and by (A.A.V.O) in the AD2 group.

Standardized neuropsychological tests were used to assess different cognitive functions. Global cognition was assessed using the Brazilian versions of the MMSE^21^ and DRS.^24^

The 15-item version of the Boston Naming Test (Consortium to Establish a Registry for Alzheimer's Disease),^32^ along with the Animals fluency^33^ test, was used for testing semantic recall while the word fluency test (FAS) was applied to assess verbal fluency.^34^ Short-term memory was evaluated using the subtest digit forward (DRS). Finally, the Clock Drawing Test (CDT)^35^ was also used to evaluate executive and attention functions whereas the digits backward (DRS) subtest was applied to evaluate working memory.

**Data analysis.** Between-group comparisons of demographic variables (age, schooling, gender) were made using one-way analyses of variance and Bonferroni *post hoc* tests. In order to evaluate the clinical data, t tests for independent samples (AD groups) were performed for each test. The severity of dementia was defined by the Dementia Rating Scale, history of progressive cognitive impairment confirmed by the patient caregiver using the IQCODE, Neuropsychiatric Inventory and by Cornell Depression Scale in Dementia scores (CSDD).

Additionally, one-way ANOVAs and Bonferroni *post-hoc* tests were used to compare mean scores on each neuropsychological test across all three groups.

## RESULTS

**Between-group comparisons of demographic and clinical characteristics.** One-way ANOVAs comparing patient and control groups showed that subjects in the AD1 group were older [F(2.93)=10.77; p=0.004] and had less formal education [F(2.95)=14.80; p<0.001] than those in both AD2 and Control groups.

The two patient groups had similar severity of dementia [CDR score, t(54)= −1.86; p=0.68], similar scores on the IQCODE [t(54)= −2.44; p=0.42], but differed for neuropsychiatric symptoms [NPI score, t(54)= 0.86; p<0.001] and signs of depression [CDSD score, t(54)=3.06; p<0.001]. Thus, the AD1 group showed higher scores on both the NPI and CDSD than the AD2 group. Table 1 summarizes the demographic and clinical characteristics of the study groups.

**Table 1:** Demographic and clinical characteristics of study subjects.

	AD1 (n=22)	AD2 (n=34)	EC (n=40)
Females/Males	15/7	31/3	24/16
Mean age	78.27±6.70*	72.56±4.09	71.10±6.72
Mean schooling	6.73±4.00*	11.47±3.33	13.25±5.57
CDR	1.25±0.57	1.53±0.50	–
IQCODE score	3.90±0.58	4.29±0.57	2.57±0.96
NPI total score	17.36±11.80	15.19±1.47	4.90±6.53
CDSD total score	10.14±6.81	5.62±1.47	5.28±4.50

AD1: patients from Brasília-DF; AD2: patients from Palmas-TO; EC: elderly controls; CDR: Clinical Dementia Rating; IQCODE: Informant Questionnaire on Cognitive Decline in the Elderly; NPI: Neuropsychiatric Inventory; CDSD: Cornell Depression Scale in Dementia.

* Significant difference between AD1 and AD2 (p<0.001).

**Between-group comparisons of neuropsychological test scores.** Mean test scores are given in Table 2. Separate one-way ANOVAs and *post hoc* analysis showed significant between-group differences on the neuropsychological tests, with the exception of DRS Construction [F(2.93)= 2.42; p=0.094].

**Table 2:** Mean neuropsychological test scores in patients and controls.

		AD1	AD2	Comparison between AD1 and AD2 (p values)	EC
**Global cognition**	MMSE	17.95±4.19	20.53±2.56	0.16	27.03±6.42 (0-30)
	DRS-Total	112.82±8.59	98.85±10.41	0.007	136.15±22.37 (0-144)
**Memory**	DRS-Memory	12.09±3.72	22.38±1.25	<0.001	23.33±3.97 (0-25)
	DRS-Digit Span Forward	5.05±1.36	2.18±1.24	<0.001	6.60±1.49 (0-8)
**Attention and executive function**	DRS-Attention	34.00±2.41	30.09±3.89	0.007	35.25±5.81 (0-37)
	DRS-Initiation/Perseveration	24.59±4.80	14.38±1.20	<0.001	34.80±6.00 (0-37)
	DRS-Digit Span Backward	2.64±1.36	0.97±1.56	<0.001	4.28±1.39 (0-8)
	Clock Drawing Test-Part 1	4.59±2.88	3.41±1.45	0.21	8.60±2.64 (0-10)
**Language ability**	Boston Naming Test	12.59±2.15	15.00±0.00	<0.001	14.50±2.40 (0-15)
	Letter Fluency (FAS)	16.50±9.95	37.65±7.14	<0.001	35.55±13.78
	Category Fluency (Animals)	5.59±2.68	13.41±2.43	<0.001	17.18±5.42
**Abstract concept formation**	DRS-Conceptualization	36.27±2.22	27.47±7.17	<0.001	37.03±6.16 (0-39)
**Visuospatial ability**	DRS-Construction	5.86±0.64	4.53±4.31	0.21	5.75±1.12 (0-6)
	Clock Drawing Test-Part 2	7.45±2.48	4.47±1.89	<0.001	9.28±2.21 (0-10)

AD1: patients from Brasília-DF; AD2: patients from Palmas-TO; EC: elderly controls. Higher test scores indicate better performances. In the elderly control column, ranges of test scores are reported in brackets except for tests with no maximum established score (i.e., Word ﬂuency FAS, and Animals).

*Post hoc* Bonferroni tests revealed that both patient groups were significantly impaired compared with the control group on all tests (p<0.001). However, the patient groups showed different mean scores on each of the tests, except for the Clock Drawing Test-part 1 (p=0.21). Thus, the AD1 group was characterized by relatively significant deficits in recall (DRS-Memory), semantic memory (Boston Naming Test) and verbal fluency. On the other hand, the AD2 group showed lower scores on attention, executive functioning and working memory.

It is noteworthy that the AD group from Brasília showed higher scores on the Cornell Depression Scale in Dementia^31^ compared with the AD2 and Elderly Control groups.

## DISCUSSION

The present study explored the neuropsychological characteristics of one group of patients diagnosed with AD from Palmas and another from Brasília by comparing them with a control group of healthy elderly.

Concerning global cognitive abilities, results on the MMSE test and DRS scale (total score) showed the presence of substantial cognitive deficits in both AD groups. However, the scores were lower for the Palmas group compared to the Brasília group considering the cut-off point.

In-depth analysis revealed that the DRS scores of the AD2 group showed low performance in attention (DRS-Attention), abstract verbal concept formation and associative thinking (DRS-Conceptualization) as well as in executive functions (DRS-IP).

It is worth mentioning that, although AD2 patients had low scores on the DRS-IP, normal scores were observed for Supermarket items, suggesting preservation of verbal fluency and initiation. These results were consistent with those for Verbal Fluency-FAS and Animals (similar scores compared to EC) where AD2 subjects showed adequate performance on both tests, in contrast to the performance observed for the AD1 group.

The low score observed for DRS-IP indicated impairments in bilateral motor planning and copying repetitive geometric patterns. Poor performance in copying five geometric figures of varying difficulty, and the writing of one's name (DRS-Construction), under both drawing-to-command and copying a model conditions (Clock Drawing Test) are suggestive of visuospatial and visuoconstructive deficits^35^ as well as working memory impairment (DRS-Digit Span Backward).

Surprisingly however, the AD2 group obtained high scores on the DRS-Memory subscale (mean score: 22.38 ±1.25; cut-off point: 22) suggesting preservation of episodic memory, as well as orientation for time, place and current events. Additionally, on the Boston naming test, these patients received full credit for uncued naming responses as well as for the correct naming of drawings in response to stimulus (semantic) cues.

The literature shows that Alzheimer's disease is characterized by prominent amnesia with additional deficits in language and semantic knowledge, abstract reasoning, executive functions, attention, and visuospatial abilities.^7^ Patients from the AD2 group showed impairments in some cognitive abilities, but we failed to find neuropsychological evidence indicating episodic memory impairment.

The mean educational level of the AD2 group was similar to the average schooling for the EC, but was higher than that observed in the AD1 group. This difference could be one explanation for the low neuropsychological performance observed in the AD1 group considering the established association of AD with lower educational attainment. However, these differences in educational level do not explain the striking discrepancies in cognitive profile between the AD groups.

Additionally, although both AD groups had CDR scores of greater than 1, and their caregivers reported decline on the IQCODE, these results were incongruent in the AD2 group. The diagnosis of AD strictly hinges on identifying memory and other cognitive domain involvement that lead to functional decline and impairment in activities of daily living. Thus, since the patients obtained high scores on memory tests, we are led to assume an overestimate of their deficits by caregivers/relatives.

In general, the results called our attention to the composition of the sample from Palmas and led us to question the diagnostic accuracy of these patients. Did these patients have Alzheimer's disease? Or did they have another type of dementia? Are there any sociodemographic variables influencing the results that may have caused the differences between the groups from Palmas and Brasília?

From this perspective, an appropriate and comprehensive neuropsychological assessment is recommended in order to obtain a detailed cognitive profile that allows a more accurate diagnosis to be reached. Additionally, we consider it important to assess the presence of subjective complaints or anosognosia symptoms; and collect information carefully about daily living activities, that is, to assess what spheres of daily living are most impacted by the disease.

To this end, a multidisciplinary approach and proper qualification and training for health care professionals all play a crucial role. It is important to develop the ability to adequately capture the range of variability observed in patients with cognitive impairments, to understand the nature of the disease, and to acquire the ability to accurately assess, define and diagnose the transitional states between normal aging, AD, and other dementias.

This work has some limitations. First, the study design is descriptive and not longitudinal, and was thus able to provide only a snapshot view of the current stage of these patients. For this reason, a follow-up study involving AD2 patients would be desirable in order to see whether they progress to a specific type of dementia syndrome or to AD.

Second, unfortunately the groups were not equally matched for age and schooling level, a factor which may have influenced the interpretation of the data.

Third, some specific cognitive domains, such as language and specific types of memory, were not directly assessed in this study and neither was functional profile using a specific measure. Therefore, further work with the AD2 group is clearly needed to define a more accurate and complete understanding of the cognitive profile of these patients taking into consideration the limitations described above.

Fourth, the comparison between the AD samples was carried out based on neuropsychological scores. Other information such as data collected from anamnesis, neurological examination and laboratory and neuroimaging exams was not included.

Finally, the evaluation of the patients was performed by two different neuropsychologists/researchers and therefore differences between the evaluators cannot be ruled out. Researchers were not blinded to information about the previous diagnoses performed by the Neurologists.

## Figures and Tables

**Figure 1:**
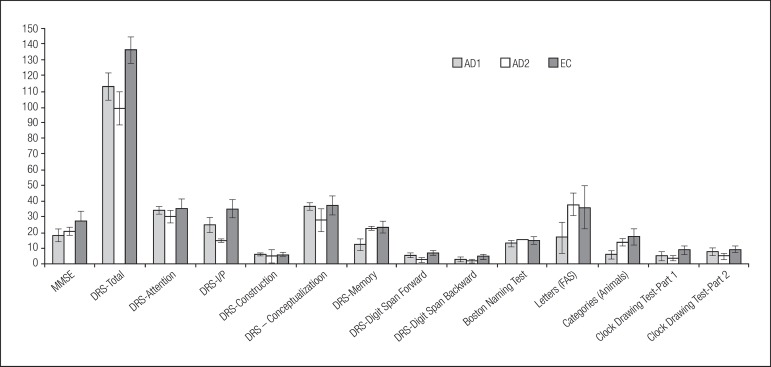
Mean (and SE bars) for neuropsychological test scores for patients from Brasília-DF (AD1), patients from Palmas-TO (AD2), and elderly controls (EC).
